# Reviewed article: Proença R, Nunes CCMN, Romano APM, Silva CP, Frank CHM, Moraes FS, Gonçalves FSS, Bandeira GS, Rodrigues IA, Cavalcante JR, Fonseca JLP, Silva LC, Duarte MMS, Maia MA, Mello MS, Nascimento MP, Nienov OH, Costa POM, Martins RCC, Melo SA, Ferreira VP, Casaroto VS, Freitas DRC, Almeida TVR, Garcia MHO, Barreira Filho EB. National Guidelines for Public Health Emergency Surveillance within the Brazilian Unified Health System. Epidemiol Serv Saude. 2026:35;e20260071

**DOI:** 10.1590/S2237-96222026v35e20260071.c

**Published:** 2026-10-12

**Authors:** Mariana Del Grossi Moura

**Affiliations:** 1 Universidade de Sorocaba, Sorocaba, SP, Brazil

## First round comments

Prezados autores,

Agradecemos a submissão do manuscrito RESS-2026-0071 intitulado “Diretrizes para Vigilância das Emergências em Saúde Pública” à Revista Epidemiologia e Serviços de Saúde: revista do SUS (RESS).

Após a revisão do seu artigo, identificamos a necessidade de uma revisão maior para que o manuscrito esteja de acordo com os padrões editoriais da revista e para viabilizar sua publicação.

Além das contribuições detalhadas pelos revisores, apresento a seguir orientações complementares que considero importantes para o aprimoramento do manuscrito:

1. O manuscrito apresenta-se como diretriz técnica voltada à organização da vigilância das emergências em saúde pública no âmbito do SUS. Como sugestão de aprimoramento do relato, recomenda-se considerar as orientações do RIGHT Statement (Reporting Items for Practice Guidelines in Healthcare), guia internacional para transparência na apresentação de diretrizes em saúde, indexado na Rede EQUATOR. Os autores podem revisar o resumo e o texto completo à luz do checklist RIGHT, verificando se estão descritos, quando aplicável: escopo, objetivo e população-alvo; processo de identificação e utilização das evidências; método de formulação das recomendações; composição do grupo elaborador e eventual validação externa; declaração de financiamento e conflitos de interesse; versão, data e plano de atualização; e considerações sobre aplicabilidade no contexto do Sistema Único de Saúde;

2. Título: explicitar no título que se trata de diretrizes nacionais e indicar o contexto (Brasil ou Sistema Único de Saúde). Exemplo: Diretrizes nacionais para vigilância das emergências em saúde pública no âmbito do Sistema Único de Saúde (SUS);

3. Resumo: avaliar a possibilidade de simplificar a estrutura do resumo, mantendo os blocos “Objetivo - Diretrizes - Conclusão”, com eventual incorporação do conteúdo atualmente apresentado em “Discussão” ao corpo das diretrizes ou à conclusão. Essa adequação pode contribuir para maior objetividade e padronização do resumo, sem prejuízo do conteúdo;

4. Palavras-chave: ajustar na formatação do campo. A expressão deve ser grafada em letra minúscula, seguida de dois pontos;

5. Texto completo: rever quantidade de palavras do manuscrito (deve ter 3.500 palavras da Introdução até considerações finais), pois o texto ultrapassa o limite permitido.

6. Introdução: há repetição frequente da expressão “emergências em saúde pública” ao longo da introdução. Embora não seja um erro, a repetição excessiva pode deixar o texto pouco fluido. Recomenda-se revisar a redação para evitar repetições desnecessárias, utilizando termos equivalentes ou pronomes de retomada (por exemplo: “essas emergências”, “tais eventos”, “esse contexto”), mantendo a precisão do conteúdo;

7. Revisar em todo manuscrito: adequação da forma de citação das referências no texto. As referências devem ser citadas em sistema numérico, segundo a ordem de aparecimento, com os números grafados entre parênteses e posicionados imediatamente após a passagem do texto a que se referem, antes da pontuação correspondente;

8. Diretrizes: observa-se variação na forma de apresentação das subseções, com alternância entre descrição conceitual, explicação operacional e exemplos práticos. Como sugestão de aprimoramento, recomenda-se organizar, quando aplicável, em sequência lógica como: definição/conceito; objetivo da estratégia; operacionalização (como implementar); e responsabilidades institucionais. Essa padronização pode favorecer maior clareza, comparabilidade entre estratégias e aplicabilidade prática do documento;

9. Considerando a complexidade e a multiplicidade de estratégias apresentadas na seção “Diretrizes”, sugere-se avaliar a possibilidade de incluir uma síntese visual que articule as principais estratégias e seus componentes. A representação gráfica pode favorecer maior clareza e facilitar a compreensão da estrutura geral da vigilância das emergências;

10. Diretrizes: alguns trechos apresentam períodos extensos e múltiplas orações coordenadas, dificultando leitura e aplicabilidade prática. Sugere-se revisar períodos excessivamente longos, segmentando-os quando possível, com vistas a melhorar clareza e fluidez;

11. Foram identificados pequenos erros de digitação e concordância ao longo do texto. Exemplos: “vigilância de epidemiológica”, “relacionados potenciais emergências”, “setor saude”. Alem disso, o uso de tempo verbal inadequado como, “precisavam ser verificados”, “eram enviadas”. Recomenda-se revisão linguística cuidadosa antes da versão final, com atenção à concordância, ortografia, pontuação e padronização dos tempos verbais;

12. DISCUSSÃO/APLICABILIDADE AO SUS: avaliar a adequação do título da seção ser intitulado apenas “Aplicabilidade ao SUS”; O primeiro parágrafo apresenta período longo, com enumeração extensa separada por ponto e vírgula. Recomendável segmentação para melhorar clareza;

13. Considerações finais: A seção está coerente com o objetivo do manuscrito e reforça adequadamente a importância da equidade. Como sugestão de aprimoramento, recomenda-se incluir breve síntese dos principais componentes apresentados ao longo das diretrizes (como as estratégias de vigilância, o monitoramento e as medidas de preparação e resposta), de modo a reforçar de forma mais explícita o atendimento ao objetivo de apresentar as diretrizes nacionais;

14. Título das tabelas: recomenda-se tornar os títulos das tabelas mais informativos e autossuficientes, permitindo sua compreensão sem necessidade de consulta ao texto, com explicitação do contexto da diretriz e do âmbito do SUS, quando aplicável;

15. Tabelas 1,2,3: recomenda-se sintetizar as informações, priorizando frases mais curtas e objetivas. Quando possível, organizar conteúdos em estruturas mais diretas, evitando construções longas;

16. Aspectos éticos: Conforme as Instruções aos Autores da RESS, as informações relativas à aprovação ética devem constar na folha de rosto. incluir na folha de rosto quadro específico contendo: (i) nome do Comitê de Ética em Pesquisa, (ii) número do parecer, (iii) data de aprovação, (iv) CAEE e (v) informação sobre o Termo de Consentimento Livre e Esclarecido, quando aplicável. Caso o estudo não tenha sido submetido à apreciação ética, é necessário justificar de forma sucinta. Remover o tópico “Aspectos éticos” do corpo do manuscrito, evitando duplicidade de informação;

17. Uso de inteligência artificial generativa: Incluir após “disponibilidade de dados”, declaração sobre o uso ou não uso de ferramentas de inteligência artificial generativa na elaboração do manuscrito.

Solicitamos que todos os comentários da revisão por pares sejam respondidos pontualmente por meio de uma carta resposta detalhada, explicando as modificações realizadas ou justificando, quando necessário, a manutenção do texto original. A nova versão do manuscrito será reconsiderada após essa reformulação e poderá passar por nova rodada de avaliação.

Atenciosamente, 

Recommendation: Major revision

## Second round comments

Prezados(as) autores(as),

Agradeço o envio da versão revisada do manuscrito. O manuscrito apresentou avanço substancial após a revisão, com incorporação da maior parte das recomendações editoriais e aprimoramento da organização geral do texto.

Antes da decisão editorial final, solicito atenção aos seguintes ajustes:

• Aspectos éticos: Conforme as Instruções aos Autores da RESS para a modalidade Diretriz, as informações relativas aos aspectos éticos devem constar em quadro específico posicionado após o resumo e as palavras-chave, e não dispersas no corpo do manuscrito. Solicita-se verificar a adequação desse item, incluindo, quando aplicável: (i) nome do Comitê de Ética em Pesquisa; (ii) número do parecer; (iii) data de aprovação; (iv) número do CAAE; e (v) informação sobre o Termo de Consentimento Livre e Esclarecido. Caso o manuscrito não tenha sido submetido à apreciação ética, recomenda-se apresentar justificativa sucinta no referido quadro, conforme orientações da RESS;

• Adequar o sistema de citações ao padrão adotado pela RESS, utilizando colchetes [ ]. Peço desculpas pela orientação anterior;

• Ajustar a formatação das palavras-chave, utilizando a primeira letra de cada palavra principal em maiúscula. Exemplo: Emergências em Saúde Pública; Saúde Pública;

• Realizar revisão linguística final do texto, com atenção à ortografia, concordância, pontuação e fluidez da redação. Como exemplos, foram identificadas expressões como “vigilância de epidemiológica”, “situações risco de desassistência”, “Esta vígilância” e “além potenciais desastres”, além de outras pequenas inconsistências que podem ser corrigidas em uma revisão geral do manuscrito;

• Verificar a extensão do manuscrito, uma vez que a versão atual apresenta 3.571 palavras da Introdução às Considerações Finais, ultrapassando discretamente o limite de 3.500 palavras estabelecido para a modalidade Diretriz.

Solicitamos que todos os comentários da revisão por pares sejam respondidos pontualmente por meio de uma carta resposta detalhada, explicando as modificações realizadas ou justificando, quando necessário, a manutenção do texto original.

Atenciosamente 

**Recommendation: **Minor revision

